# Hearing Loss and Communication Difficulty in Hospital and Hemodialysis Care Settings

**DOI:** 10.1001/jamanetworkopen.2026.8504

**Published:** 2026-04-28

**Authors:** Meaghan Lunney, Natasha Wiebe, Alex DeBusschere, Susan Szigety, Gillian Crysdale, Sonja Reid, Nancy Verdin, Stephanie Thompson, David Nicholas, Kara Schick-Makaroff, Jayna Holroyd-Leduc, Maoliosa Donald, Sharon Straus, Lorienne Jenstad, Patti-Jo Sullivan, Tanis Howarth, Julie Evans, Marcello Tonelli

**Affiliations:** 1 Department of Medicine, Cumming School of Medicine, University of Calgary, Calgary, Alberta, Canada; 2 Department of Medicine, Faculty of Medicine and Dentistry, University of Alberta, Edmonton, Alberta, Canada; 3 Faculty of Social Work, University of Calgary, Calgary, Alberta, Canada; 4 Department of Health Sciences, Faculty of Nursing, University of Alberta, Edmonton, Alberta, Canada; 5 Department of Medicine, University of Toronto, Toronto, Ontario, Canada; 6 Department of Audiology and Speech Sciences, Faculty of Medicine, University of British Columbia, Vancouver, British Columbia, Canada; 7 Alberta Health Services, Calgary, Alberta, Canada

## Abstract

This cross-sectional study evaluates the prevalence of hearing loss among specific patient populations in Alberta and explores participant perceptions about patient-health care worker communication and potential solutions.

## Introduction

People with hearing loss often experience communication barriers during health care encounters,^1^ impacting their experience and health outcomes. Communication tools and strategies exist; however, they are rarely used in practice.^2^ This care gap is likely, in part, due to low awareness about the prevalence of hearing loss in clinical settings and how to accommodate it. We did a cross-sectional study to estimate how common hearing loss is among specific patient populations in Alberta and explore participant perceptions about patient-health care worker (HCW) communication and potential solutions.

## Methods

This study follows the Strengthening the Reporting of Observational Studies in Epidemiology (STROBE) reporting guideline for cross-sectional studies^3^ and was approved by the University of Calgary health ethics research board. We enrolled adult patients receiving inpatient care or outpatient hemodialysis treatment between January and November 2023. Research coordinators met with nursing staff to screen patients for eligibility. The coordinator explained the study purpose was to determine the proportion of patients with hearing loss. Nurses identified patients with sufficient cognitive and language capacity, behavioral stability, and clinical readiness to participate. Eligible patients were informed about the study by the nurse and if interested, were approached by the coordinator, who explained the study as previously stated. Patients who provided written consent completed a hearing test through a mobile application (hearWHO),^4^ unless they self-reported use of a hearing device (thus identified as having hearing loss). After the test, participants completed a questionnaire about hearing health, sociodemographics, difficulty communicating with HCWs, and solutions.

We planned to accrue 200 inpatients and 171 outpatients, assuming a true population prevalence of 50% (margin of error, 7.5%). We reported descriptive statistics as counts and percentages and means and SDs. We used 2-sided χ^2^ and linear regression tests, respectively, to assess for differences between groups with a significance threshold of .05. We standardized by age and retested for differences using weighted generalized linear regression. We did all analyses in Stata/MP version 18.0 (StataCorp).

## Results

From 1330 screened patients, 823 were ineligible and 129 declined to participate. Six were excluded as they did not complete the hearing test or use a hearing device. A total of 372 participants were included (Table); 200 inpatients and 172 hemodialysis patients. Of the 372 participants, 213 (57.3%) were male and the median (IQR) age was 65 (52-75) years . A total of 171 had hearing loss, yielding an overall sample prevalence of 46.0% (95% CI, 40.8%-51.2%). Prevalence was not significantly different between inpatient (46.5%; 95% CI, 39.4%-53.7%) and hemodialysis (45.3%; 95% CI, 37.8%-53.1%) groups.

**Table.** Demographic, Social, and Self-Reported Hearing Health Characteristics by Hearing Loss

Characteristic	Participants, No. (%)^a^		*P* value	*P* value standardized for age^b^
	Hearing loss (n = 171)	No hearing loss (n = 201)		
Index hospital stay, d, mean (SD)	33 (55)	31 (57)	.73	.35
Readmission	4 (4.3)	14 (13.1)	.03	.16
Age, y, mean (SD)	70 (14)	57 (15)	<.001	NA
Sex				
Female	75 (43.9)	84 (41.8)	.69	.39
Male	96 (56.1)	117 (58.2)		
Preferred not to answer	0	0		
Gender				
Man	85 (53.9)	114 (57.6)	.35	.77
Woman	76 (46.1)	81 (40.9)		
Other gender	0	2 (1.0)		
Prefer not to answer	0	1 (0.5)		
Prefers a language translator	12 (7.2)	6 (3.0)	.07	.11
Household			.02	.44
With someone else	88 (54.0)	130 (67.0)	NA	NA
Alone	50 (30.7)	46 (23.7)	NA	NA
In facility care	24 (14.7)	14 (7.2)	NA	NA
Unhoused	1 (0.6)	4 (2.1)	NA	NA
Difficulty making ends meet	26 (16.8)	53 (28.0)	.01	.30
Difficulty filling prescriptions due to costs	12 (7.5)	16 (8.1)	.84	.63
Difficulty attending appointments due to transportation costs	11 (6.9)	16 (8.2)	.66	.68
Self-reported difficulties				
Vision	55 (34.4)	51 (25.9)	.08	.09
Walking	99 (61.9)	91 (46.2)	.003	.13
Climbing stairs	95 (59.4)	101 (51.3)	.13	.80
Remembering or concentrating	56 (35.0)	59 (29.9)	.31	.47
Self-care	41 (25.6)	39 (19.8)	.19	.72
Communicating	18 (11.2)	19 (9.6)	.62	.90
Loud sound exposure				
At work	77 (46.7)	99 (51.0)	.41	.45
Outside of work	43 (26.4)	66 (34.2)	.11	.61
Immediate family history (present since ≤12 y)	17 (10.9)	15 (8.0)	.35	.06

Abbreviation: NA, not applicable.

^a^ Missingness ranged from 0.0% to 7.5%.

^b^ Several differences in functional and sensory limitations between participants with and without hearing loss likely reflect age-related comorbidity rather than independent associations with hearing loss. Consistent with this, many between-group differences were attenuated after age standardization.

Participants with hearing loss expressed more difficulty communicating with HCWs (31 participants [18.6%]) compared with those with no hearing loss (11 participants [5.6%]) (Figure). The 4 most frequent suggestions for improving communication among participants with communication difficulty (regardless of hearing level) were asking patients about hearing difficulties, involving family and/or friends, using transparent masks, and rephrasing.

**Figure.**
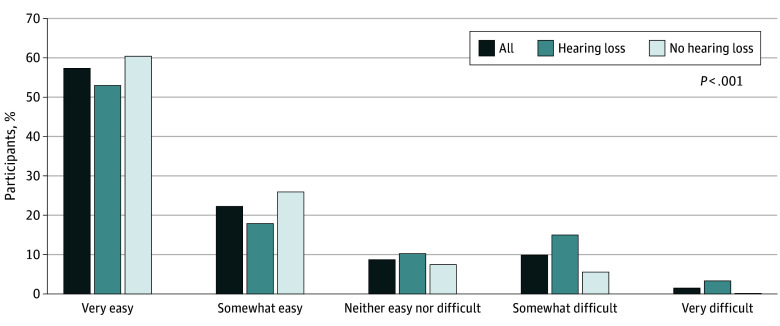
Bar Chart Showing Patient-Reported Difficulty Understanding Health Care Practitioners (N = 364)

## Discussion

Nearly half of participants in this study sample had hearing loss. Participants with hearing loss reported more difficulty communicating with HCWs than those with normal hearing. Given that hearing loss affected a substantial proportion of participants and was associated with patient-HCW communication, raising awareness about how to identify and accommodate patients with hearing loss in health care encounters is important. Not doing so may result in reduced satisfaction with communication,^5^ more misdiagnoses, increased falls, longer length of stay, complications, lower postdischarge adherence, and more readmissions.^6^

Because enrollment required clinical and behavioral stability, capacity to consent, English proficiency, and willingness to participate, these findings should be interpreted as the prevalence within the study sample, not as the population prevalence. Nonetheless, hearing loss was identified frequently enough among participants that clinicians should routinely identify and accommodate patients’ hearing needs, particularly given the associated difficulty with understanding health care information. HCW training, clear documentation practices, and a variety of effective communication tools and strategies are essential for ensuring equity and the safety of this patient population.

## Notes

Supplement. Data Sharing Statement
