# Supplementary material for: Hearing Loss and Communication Difficulty in Hospital and Hemodialysis Care Settings
Source: JAMA Netw Open. 2026 Apr 28;9(4):e268504. doi: 10.1001/jamanetworkopen.2026.8504 (PMC13126209; doi:10.1001/jamanetworkopen.2026.8504)
Supplement: Supplement. — Data Sharing Statement [file jamanetwopen-e268504-s001.pdf]

Data Sharing Statement

Lunney. Hearing Loss and Communication Difficulty in Hospital and Hemodialysis Care Settings. *JAMA Netw Open*. Published April 28, 2026.
doi:10.1001/jamanetworkopen.2026.8504

Data
Data available: No
